# Factors associated with the frequency of monitoring of liver enzymes, renal function and lipid laboratory markers among individuals initiating combination antiretroviral therapy: a cohort study

**DOI:** 10.1186/s12879-015-1206-3

**Published:** 2015-10-26

**Authors:** Jennifer Gillis, Ahmed M Bayoumi, Ann N Burchell, Curtis Cooper, Marina B Klein, Mona Loutfy, Nima Machouf, Julio SG Montaner, Chris Tsoukas, Robert S Hogg, Janet Raboud, The Canadian Observational Cohort (CANOC) collaboration

**Affiliations:** Toronto General Research Institute, University Health Network, Toronto, Canada; University of Toronto, Toronto, Canada; Centre for Research on Inner City Health, Li Ka Shing Knowledge Institute, St. Michael’s Hospital, Toronto, Canada; Li Ka Shing Knowledge Institute, St. Michael’s Hospital, Toronto, Canada; University of Ottawa, The Ottawa Hospital Research Institute, Ottawa, Canada; McGill University Health Centre, McGill University, Montreal, Canada; Women’s College Research Institute, Toronto, Canada; Maple Leaf Medical Clinic, Toronto, Canada; Clinique Médicale l’Actuel, Montreal, Canada; British Columbia Centre for Excellence in HIV/AIDS, Vancouver, Canada; Simon Fraser University, Burnaby, Canada; Department of Family and Community Medicine, St. Michael’s Hospital, Toronto, Ontario Canada

**Keywords:** HIV, Antiretroviral therapy, Laboratory markers, Liver enzymes, Renal function, Lipids, Clinical monitoring

## Abstract

**Background:**

As the average age of the HIV-positive population increases, there is increasing need to monitor patients for the development of comorbidities as well as for drug toxicities.

**Methods:**

We examined factors associated with the frequency of measurement of liver enzymes, renal function tests, and lipid levels among participants of the Canadian Observational Cohort (CANOC) collaboration which follows people who initiated HIV antiretroviral therapy in 2000 or later. We used zero-inflated negative binomial regression models to examine the associations of demographic and clinical characteristics with the rates of measurement during follow-up. Generalized estimating equations with a logit link were used to examine factors associated with gaps of 12 months or more between measurements.

**Results:**

Electronic laboratory data were available for 3940 of 7718 CANOC participants. The median duration of electronic follow-up was 3.5 years. The median (interquartile) rates of tests per year were 2.76 (1.60, 3.73), 2.55 (1.44, 3.38) and 1.42 (0.50, 2.52) for liver, renal and lipid parameters, respectively. In multivariable zero-inflated negative binomial regression models, individuals infected through injection drug use (IDU) were significantly less likely to have any measurements. Among participants with at least one measurement, rates of measurement of liver, renal and lipid tests were significantly lower for younger individuals and Aboriginal Peoples. Hepatitis C co-infected individuals with a history of IDU had lower rates of measurement and were at greater risk of having 12 month gaps between measurements.

**Conclusions:**

Hepatitis C co-infected participants infected through IDU were at increased risk of gaps in testing, despite publicly funded health care and increased risk of comorbid conditions. This should be taken into consideration in analyses examining factors associated with outcomes based on laboratory parameters.

## Background

Comorbidities are of particular concern in HIV-positive populations as successful treatment of HIV with combination antiretroviral therapy (cART) and increased incident HIV infection in older individuals have led to an increase in the average age of HIV-positive populations [1, 2]. Laboratory measurement of liver enzymes, renal function, and lipid levels in HIV-positive individuals is essential for detecting early development of such comorbidities, and for identification of treatment-related toxicities and adverse interactions between antiretroviral and non-antiretroviral medications [3].

The recommended frequency of laboratory assessment after cART initiation varies among HIV treatment guidelines [4–7]. To monitor for potential toxicity related to cART, all guidelines recommend laboratory assessment of liver enzymes, renal function, and lipid levels at entry to care and prior to initiation of cART, and at least annually thereafter. The Department of Health and Human Services (DHHS) [4] and British HIV Association (BHIVA) [6] recommend the most frequent monitoring of liver enzymes, at least every 3-6 months. The British Columbia Centre for Excellence in HIV/AIDS (BC CfE) [7] recommends the most frequent monitoring of renal function, every 3-4 months.

Risk factors and clinical features also influence the recommended frequency of testing within the guidelines. For instance, BHIVA recommends more frequent measurement of lipid levels for patients at high risk of cardiovascular disease [6, 8]. Similarly, European AIDS Clinical Society (EACS) and BC CfE guidelines suggest more frequent monitoring of liver enzymes and renal function for patients taking hepatotoxic or nephrotoxic drugs, or patients with elevated risk of liver or kidney disease due to factors such as hepatitis C co-infection and diabetes [5, 9].

We conducted this analysis to determine whether demographic and clinical characteristics were associated with rates of laboratory testing and clinically significant gaps in measurement of liver enzymes, renal function, and lipid tests among individuals who initiated cART since January 1, 2000 in Canada. The motivation for this work was primarily to determine if HIV-positive individuals were accessing services equitably within a publicly funded health care system and, secondly, to determine whether there was evidence of measurement bias that could aid in interpretation of analyses of HIV co-morbidities that rely upon routinely collected laboratory marker data.

## Methods

### Study population

The Canadian Observational Cohort (CANOC) is a national collaboration of eight cohorts of antiretroviral-naive HIV-positive individuals initiating cART after January 1, 2000 from three Canadian provinces (British Columbia (BC), Quebec, and Ontario). The study design has been reported previously [10]. Briefly, to be eligible for CANOC, patients must have documented HIV infection, reside in Canada, be at least 18 years of age, initiate their first antiretroviral regimen comprised of at least three agents, and have at least one measurement of HIV plasma viral load and CD4 cell count within six months of initiating cART. Individuals under follow-up at sites and for time periods with electronic laboratory data were included in this analysis. Time periods for which electronic laboratory data were available for each site were determined through consultation with site investigators. Patient selection and data extraction were performed locally at the data centers of the participating cohort studies. Sites were instructed to provide all available data on a predefined set of demographic, laboratory, and clinical variables. Non-nominal data from each cohort were then pooled at the Project Data Centre in Vancouver, BC.

### Ethics statement

The human subjects activities of CANOC have been approved by the Simon Fraser University Research Ethics Board (REB) and the University of British Columbia REB. Additionally, approval from local institutional review boards (IRBs) was granted at each participating cohort site, as follows: Providence Health Care Research Institute Office of Research Services, the Ottawa Hospital REB, University Health Network (UHN) REB, Véritas IRB, Biomedical C REB of the McGill University Heath Centre (MUHC), University of Toronto HIV REB, and Women’s College Hospital REB.

Local cohorts have obtained written consent from participants with the following exceptions: HAART Observational Medical Evaluation and Research (HOMER) Cohort (IRB approves the retrospective use of anonymous administrative data without requiring consent; an information sheet is provided in lieu of a consent form); Ottawa Hospital Cohort (IRB approves the anonymous use of data retrospectively abstracted from clinical care databases without requiring consent); UHN (REB approves the anonymous use of data retrospectively abstracted from clinical care databases without requiring consent); MUHC (IRB approves the anonymous use of data retrospectively abstracted from clinical care databases without requiring consent; patients sign a general waiver on opening a medical chart at the hospital but no specific study related consent); Maple Leaf Medical Clinic (REB has approved the anonymous use of data retrospectively abstracted from clinical care databases without requiring consent); Electronic Antiretroviral Therapy (EARTH) Cohort (REB approves the anonymous use of data retrospectively abstracted from clinical care databases without requiring consent; patients sign a general waiver on opening a medical chart at the hospital but no specific study related consent).

### Outcome measures

The three sets of laboratory measurements studied were (a) the liver enzymes alanine transaminase (ALT) or aspartate transaminase (AST), (b) creatinine, as a measure of renal function, and (c) at least one of the following measures of lipid levels: LDL or HDL cholesterol, triglycerides or total cholesterol. For each set of measurements, we determined the rate of measurement per year (i.e., the number of tests per person-year of observation) and whether the gaps between pairs of consecutive measurements exceeded 12 months, which was considered to be a clinically important gap between measurements. To avoid inflated rates of measurement from testing during apparent hospitalization or intensive monitoring during changes in cART or other therapies (e.g. for hepatitis C virus), repeat measurements within 30 days were excluded from determination of the rates of measurement.

### Explanatory variables of interest

We selected potential prognostic factors for laboratory testing based on a priori knowledge of associations with frequency of clinical follow-up. We examined demographic and clinical characteristics including age, sex, province, race, HIV risk factors such as injection drug use (IDU) and men having sex with men (MSM), year of first HIV positive test, co-infection with hepatitis B or C, CD4 count and HIV viral load at cART initiation, and class of ARV in the initial cART regimen. Grade 3 or 4 elevations for each laboratory measure were defined as follows: AST (5 times upper limit normal (ULN) = 170 units per litre (U/L)), ALT (5 times ULN * =* 200 U/L), creatinine (3 times ULN* =* 330 μmol/L for men, 294 μmol/L for women), LDL (5.0 mmol/L), triglyceride (8.48 mmol/L), total cholesterol (7.78 mmol/L) and total cholesterol/HDL ratio (7.0).

### Statistical analysis

Demographic and clinical characteristics were summarized for participants who were included and excluded from the analysis using frequencies and percentages for categorical variables and medians and interquartile ranges for continuous variables. Chi square tests, Cochran-Armitage tests for trend and Wilcoxon rank sum tests were used to compare rates of measurement and the probability of a gap among subgroups of participants according to demographic or clinical characteristics.

Zero-inflated negative binomial regression models were used to examine the associations of demographic and clinical characteristics with the rates of measurement of ALT/AST, creatinine, and lipids during follow-up. The negative binomial distribution accounts for overdispersion in the distribution of the number of laboratory tests relative to a Poisson distribution, while a zero-inflated model allows for the explicit modeling of the probability of having no laboratory tests. Generalized estimating equation (GEE) models with a logit link were used to examine factors associated with a 12 month gap between sequential measurements. An unstructured correlation matrix was used to account for correlation among repeat observations within individuals. Time-updated variables were used to model the associations of CD4 counts, HIV viral load and grade 3 or 4 elevations of laboratory markers with a 12 month gap between measurements. Sensitivity analyses were conducted with an 18 month gap between measurements. For variables with large amounts of missing data, separate categories were created for missing values. All analyses were conducted with SAS 9.4 (SAS Institute, Cary, North Carolina, USA).

## Results

A total of 7718 participants were enrolled into CANOC as of September 2011, of whom 3940 were followed during calendar time periods when electronic laboratory data were available at their site. Clinical and demographic characteristics are described for all patients and by availability of electronic laboratory data in Table 1. The median duration of electronic laboratory follow-up was 3.5 (IQR 2.0, 6.1) years. The median age was 39 years, 83 % were male, 58 % were men who have sex with men (MSM), 17 % were IDU and 18 % were co-infected with hepatitis C.

**Table 1:** Characteristics of included and excluded participants due to availability of electronic data

Characteristics	Cohort (*n =* 7718)	Included (*n =* 3940)	Excluded (*n =* 3778)
Province			
British Columbia	3588 (46 %)	787 (20 %)	2801 (74 %)
Ontario	2394 (31 %)	1715 (44 %)	679 (18 %)
Quebec	1736 (22 %)	1438 (36 %)	298 (8 %)
Age	40 (34-47)	39 (33-46)	41 (34-48)
Male	6208 (81 %)	3255 (83 %)	2953 (78 %)
Race			
Caucasian	2046 (27 %)	1040 (26 %)	1006 (27 %)
Black	512 (7 %)	373 (9 %)	139 (4 %)
Aboriginal	484 (6 %)	117 (3 %)	367 (10 %)
Other	448 (6 %)	285 (7 %)	163 (4 %)
Unknown	4228 (55 %)	2125 (54 %)	2103 (56 %)
HIV Risk factor			
Men having sex with men	2758 (49 %)	1877 (58 %)	881 (37 %)
Injection drug use	1766 (31 %)	555 (17 %)	1211 (51 %)
From Endemic Country	684 (12 %)	553 (17 %)	131 (6 %)
Unknown	2100 (27 %)	680 (17 %)	1420 (38 %)
Year of cART initiation			
2000	546 (7 %)	271 (7 %)	275 (7 %)
2001-2005	2980 (39 %)	1723 (44 %)	1257 (33 %)
>2005	4192 (54 %)	1946 (49 %)	2246 (59 %)
Baseline cART Regimen			
NNRTI-based	3562 (46 %)	1727 (44 %)	1835 (49 %)
Boosted PI-based	3091 (40 %)	1506 (38 %)	1585 (42 %)
Other PI-based	928 (12 %)	611 (16 %)	317 (8 %)
Other	137 (2 %)	96 (2 %)	41 (1 %)
Year of HIV+ Test	2003 (2000-2006)	2003 (2000-2006)	2002 (1998-2005)
Hepatitis C positive	1922 (27 %)	663 (18 %)	1259 (37 %)
Hepatitis B positive	386 (10 %)	304 (11 %)	82 (9 %)
Baseline CD4 (cells/mm^3^)	210 (110-300)	210 (112-297)	210 (110-310)
>500 cells/mm^3^	431 (6 %)	164 (4 %)	267 (7 %)
350-500 cells/mm^3^	970 (13 %)	483 (12 %)	487 (13 %)
200-350 cells/mm^3^	2745 (36 %)	1444 (37 %)	1301 (34 %)
<200 cells/mm^3^	3572 (46 %)	1849 (47 %)	1723 (46 %)
Baseline Viral Load (log_10_ copies/mL)	4.9 (4.3-5.1)	4.9 (4.4-5.2)	4.8 (4.2-5.0)

Results are presented as median (interquartile range) or *N* (%)

Ninety-three percent of participants for whom electronic laboratory data were available had at least one ALT/AST measurement; the median rate of measurement of ALT/AST per year of follow-up was 2.8 (IQR 1.6, 3.7). The rate of measurement differed by province, gender, age, race, HIV risk factor, baseline regimen, and IDU history and hepatitis C co-infection (Table 2). Hepatitis C co-infected participants with a history of IDU had a lower rate of ALT/AST measurement than other participants (median of 1.83 *versus* 2.89,3.31, and 2.90 for HCV- non-IDU participants, HCV+ non-IDUs and HCV- IDUs respectively, *p <* 0.0001, Table 2). In the multivariable zero-inflated negative binomial regression model, a history of IDU was significantly associated with a lower probability of having at least one ALT/AST measurement while under follow-up (odds ratio, OR = 0.19, 95 % confidence interval (CI) = (0.11, 0.32), *p <* 0.0001). Among participants with at least one measure of ALT/AST, Aboriginal ancestry was associated with lower rates of measurement (rate ratio (RR) =0.87, 95 % CI = (0.79, 0.96), *p <* 0.01), as was hepatitis C co-infection among participants with a history of IDU (RR = 0.79, 95 % CI = (0.74, 0.83), *p <* 0.0001) relative to HIV mono-infected non-IDUs. Increasing age (RR per 10 years =1.02, 95 % CI = (1.00, 1.04), *p =* 0.03) was associated with a higher rate of measurement of ALT/AST.

**Table 2:** Median rate of measurement and probability of having a gap greater than 12 months between measurements of liver, renal or metabolic function

	Liver (AST/ALT)		Renal (Creatinine)		Metabolic (Lipids)	
	Annual rate of testing, Median (IQR)	Subjects with ≥ 1 12-month gap	Annual rate of testing, Median (IQR)	Subjects with ≥ 1 12-month gap	Annual rate of testing, Median (IQR)	Subjects with ≥ 1 12-month gap
Overall	2.76 (1.60,3.73)	22 %	2.55 (1.44,3.38)	18 %	1.42 (0.50,2.52)	33 %
First year of cART	3 (0, 4)	--	2 (0, 4)	--	1 (0, 3)	--
Subsequent years	2.23 (0.31, 3.41)	--	1.78 (0, 3.06)	--	0.85 (0, 2.21)	--
Region						
British Columbia	3.25 (0.82,4.56)***	33 %***	1.66 (0.41,3.08)***	23 %***	1.05 (0.22,2.70)**	24 %***
Ontario	2.53 (1.37,3.53)	17 %	2.35 (1.33,3.20)	14 %	1.45 (0.50,2.46)	32 %
Quebec	2.88 (2.08,3.56)	21 %	2.88 (2.07,3.56)	17 %	1.48 (0.66,2.53)	38 %
Gender						
Male	2.80 (1.65,3.77)*	21 %***	2.59 (1.47,3.41)**	16 %**	1.49 (0.57,2.60)***	31 %***
Female	2.60 (1.39,3.56)	29 %	2.33 (1.17,3.28)	24 %	1.02 (0.33,2.09)	43 %
Age						
<55	2.73 (1.57,3.71)*	23 %**	2.52 (1.43,3.36)**	18 %**	1.39 (0.49,2.48)**	34 %*
≥55	3.03 (1.92,4.10)	12 %	2.90 (1.83,3.69)	9 %	1.83 (0.79,2.95)	23 %
Race						
Caucasian	3.03 (1.43,4.16)***	24 %***	2.49 (1.07,3.44)***	21 %***	1.64 (0.53,2.85)***	31 %**
Black	2.73 (1.55,3.74)	26 %	2.27 (1.23,3.17)	18 %	1.01 (0.43,2.09)	45 %
Aboriginal	2.07 (0.51,3.59)	45 %	1.08 (0.28,2.54)	30 %	0.45 (0.12,1.95)	29 %
Other	3.38 (2.39,4.40)	15 %	2.62 (1.72,3.49)	12 %	2.03 (0.75,2.98)	25 %
Unknown	2.64 (1.67,3.43)	20 %	2.63 (1.70,3.39)	16 %	1.41 (0.52,2.34)	34 %
Risk Factor						
MSM	2.97 (1.98,3.92)***	18 %***	2.72 (1.81,3.48)***	15 %***	1.53 (0.61,2.61)***	32 %**
Non-MSM	2.59 (1.26,3.68)	31 %	2.28 (0.97,3.32)	24 %	1.02 (0.31,2.02)	41 %
IDU	2.13 (0.74,3.66)***	38 %***	1.46 (0.41,3.02)***	29 %***	0.66 (0.00,1.63)***	34 %
Non-IDU	2.89 (1.87,3.84)	21 %	2.67 (1.79,3.48)	16 %	1.45 (0.59,2.52)	36 %
Endemic	2.75 (1.83,3.62)	22 %	2.65 (1.87,3.44)	20 %	1.21 (0.57,2.12)	50 %***
Non-Endemic	2.85 (1.63,3.86)	24 %	2.58 (1.42,3.43)	18 %	1.35 (0.46,2.46)	33 %
Unknown Risk Factor	2.40 (1.35,3.34)***	15 %***	2.33 (1.34,3.19)**	14 %*	1.91 (0.67,2.91)***	21 %***
Known Risk Factor	2.83 (1.69,3.81)	23 %	2.59 (1.46,3.43)	18 %	1.32 (0.48,2.41)	35 %
Year of cART Initiation						
>2005	2.77 (1.63,3.72)	12 %***	2.49 (1.21,3.40)*	9 %***	1.21 (0.33,2.30)***	23 %***
2001-2005	2.77 (1.60,3.79)	27 %	2.61 (1.66,3.38)	22 %	1.66 (0.63,2.72)	40 %
2000	2.53 (1.36,3.47)	53 %	2.51 (1.34,3.25)	37 %	1.45 (0.57,2.70)	49 %
Baseline CD4 count						
<200 cells/mm^3^	2.83 (1.62,3.78)***	25 %	2.55 (1.41,3.42)**	19 %	1.44 (0.56,2.59)***	34 %
200-350 cells/mm^3^	2.79 (1.71,3.67)	18 %	2.61 (1.59,3.33)	15 %	1.39 (0.52,2.47)	31 %
350-500 cells/mm^3^	2.66 (1.52,3.75)	21 %	2.50 (1.56,3.49)	17 %	1.48 (0.47,2.56)	31 %
>500 cells/mm^3^	2.07 (0.82,3.31)	28 %	1.88 (0.00,3.09)	21 %	0.88 (0.00,2.09)	34 %
Baseline Regimen						
NNRTI-Based	2.68 (1.57,3.56)**	19 %***	2.45 (1.36,3.23)*	16 %**	1.30 (0.47,2.34)***	34 %***
Boosted PI-Based	2.94 (1.59,4.01)	22 %	2.60 (1.40,3.52)	17 %	1.58 (0.54,2.73)	28 %
PI-Based	2.66 (1.58,3.51)	30 %	2.62 (1.70,3.43)	24 %	1.31 (0.42,2.36)	41 %
Other	2.95 (1.95,3.69)	18 %	2.89 (1.93,3.49)	15 %	1.97 (1.11,2.95)	40 %
Hepatitis C co-infection						
HCV+	2.38 (0.90,3.86)***	36 %***	1.82 (0.55,3.32)***	28 %***	0.88 (0.14,2.08)***	35 %
HCV-	2.82 (1.77,3.73)	19 %	2.63 (1.67,3.39)	15 %	1.52 (0.60,2.60)	32 %
Hepatitis B co-infection						
HBV+	2.73 (1.49,3.45)	17 %	2.63 (1.51,3.30)	18 %	1.41 (0.59,2.37)	35 %
HBV-	2.69 (1.74,3.55)	20 %	2.65 (1.80,3.43)	16 %	1.50 (0.59,2.50)	36 %
HCV and IDU status						
HCV- non-IDU	2.89 (1.89,3.79)***	20 %***	2.68 (1.81,3.45)***	16 %***	1.45 (0.60,2.49)***	36 %***
HCV+ non-IDU	3.31 (2.02,4.54)	25 %	3.12 (1.76,4.29)	18 %	1.78 (0.70,3.18)	34 %
HCV- IDU	2.90 (1.46,4.08)	23 %	2.30 (1.01,3.25)	15 %	1.21 (0.49,2.32)	23 %
HCV+ IDU	1.83 (0.62,3.60)	42 %	1.34 (0.27,2.93)	33 %	0.52 (0.00,1.42)	37 %
Unknown	2.45 (1.34,3.39)	17 %	2.34 (1.31,3.20)	16 %	1.75 (0.57,2.83)	24 %

*IDU* injection drug use as risk factor for HIV acquisition, *HCV*+: positive for Hepatitis C; * = <0.01, ** = <0.001, *** = <0.0001

Ninety percent and 84 % of participants had at least one measurement of creatinine and lipids, respectively. The median rates of measurement of creatinine and lipids per year of follow-up were 2.6 (IQR 1.4, 3.4) and 1.4 (IQR 0.5, 2.5), respectively. Associations of demographic and clinical variables with rates of creatinine and lipid measurements were similar to those for liver enzymes (Table 3). Participants with a history of IDU were less likely to have any creatinine or lipid measurements during the study period (RR = 0.22 and 0.17, respectively). Among participants with at least one creatinine or lipid measurement, hepatitis C co-infected IDUs had lower relative rates of creatinine and lipid measurements (RR = 0.80 and RR = 0.65, respectively) than HIV mono-infected non-IDUs. Abacavir use was associated with higher rates of lipid measurements (RR = 1.13, 95 % CI = (1.08, 1.18), *p-*value < 0.0001).

**Table 3:** Multivariable analysis of prognostic factors for rate of laboratory measurement according to zero-inflated negative binomial models

	Liver (AST/ALT)			Renal (Creatinine)			Metabolic (Lipids)		
	(*N =* 3934)			(*N =* 3530)			(*N =* 3530)		
	Rate ratio	95 % CI	*p-*value	Rate ratio	95 % CI	*p-*value	Rate ratio	95 % CI	*p-*value
Negative binomial									
Province									
British Columbia	Ref			Ref			Ref		
Ontario	0.98	(0.94,1.03)	0.48	1.35	(1.29,1.42)	<0.0001	1.13	(1.05,1.21)	<0.001
Quebec	1.20	(1.13,1.27)	<0.0001	1.70	(1.60,1.80)	<0.0001	1.45	(1.34,1.57)	<0.0001
Age (per 10 years)	1.02	(1.00,1.04)	0.03	1.03	(1.01,1.05)	<0.01	1.05	(1.02,1.08)	0.0001
Male	1.02	(0.98,1.07)	0.37	1.01	(0.96,1.06)	0.72	1.14	(1.07,1.22)	0.0001
Race									
Caucasian	Ref			Ref			Ref		
Black	1.03	(0.97,1.10)	0.37	0.93	(0.86,1.00)	0.04	0.80	(0.72,0.89)	<0.0001
Aboriginal	0.87	(0.79,0.96)	<0.01	0.87	(0.79,0.97)	<0.01	0.84	(0.72,0.97)	0.02
Other	1.11	(1.04,1.18)	<0.01	1.09	(1.02,1.16)	<0.01	1.06	(0.97,1.16)	0.19
Unknown	0.89	(0.85,0.93)	<0.0001	0.92	(0.88,0.96)	<0.001	0.76	(0.71,0.81)	<0.0001
HCV and IDU status									
HCV-non-IDU	Ref			Ref			Ref		
HCV+ non-IDU	1.05	(0.97,1.14)	0.21	1.07	(0.99,1.15)	0.09	1.00	(0.89,1.12)	0.99
HCV-IDU	0.98	(0.89,1.07)	0.62	0.95	(0.87,1.05)	0.31	0.95	(0.83,1.08)	0.43
HCV+ IDU	0.79	(0.74,0.83)	<0.0001	0.80	(0.75,0.85)	<0.0001	0.65	(0.59,0.71)	<0.0001
Unknown	0.97	(0.93,1.02)	0.21	1.00	(0.96,1.04)	0.96	1.28	(1.20,1.36)	<0.0001
CD4 measurements:									
<3 per year	Ref			Ref			Ref		
3-5 per year	1.87	(1.79,1.95)	<0.0001	1.76	(1.68,1.84)	<0.0001	1.84	(1.73,1.97)	<0.0001
≥6 per year	2.67	(2.52,2.83)	<0.0001	2.56	(2.41,2.71)	<0.0001	2.79	(2.56,3.03)	<0.0001
First cART regimen									
Other	Ref			Ref			Ref		
NNRTI based	0.95	(0.86,1.05)	0.32	0.97	(0.88,1.06)	0.47	0.78	(0.68,0.90)	<0.001
Boosted PI based	0.96	(0.87,1.07)	0.48	1.01	(0.91,1.11)	0.89	0.88	(0.77,1.02)	0.08
PI based	0.95	(0.85,1.05)	0.32	0.95	(0.86,1.05)	0.35	0.78	(0.67,0.90)	<0.001
Abacavir use	--	--	--	--	--		1.13	(1.08,1.18)	<0.0001
Tenofovir use	--	--	--	0.99	(0.96,1.03)	0.68	--	--	--
Zero-inflated									
IDU	0.19	(0.11,0.32)	<0.0001	0.22	(0.15, 0.34)	<0.0001	0.17	(0.11,0.26)	<0.0001
Unknown HIV risk factor	1.32	(0.50,3.48)	0.58	1.14	(0.59,2.19)	0.70	0.96	(0.43,2.14)	0.92

*IDU* injection drug use as risk factor for HIV acquisition, *HCV*+, positive for Hepatitis C

Twenty-two percent, 18 % and 33 % of participants had at least one 12 month gap between measurements of ALT/AST, creatinine and lipid measurements, respectively. In a multivariable GEE model with a logit link, viral load suppression (<50 copies/mL) at the start of an inter-test gap (OR = 0.67, 95 % CI = (0.59,0.77), *p <* 0.0001) and older age (OR per 10 years = 0.77, 95 % CI = (0.70, 0.84), *p <* 0.0001) were associated with decreased risk of a 12 month gap between ALT/AST measurements while hepatitis C co-infected participants with a history of IDU were at an increased risk of a 12 month gap (OR = 2.21, 95 % CI = (1.73,2.82), *p <* 0.0001) (Table 4). Similar results were observed for gaps in creatinine and lipid measurements (Table 4); however, male gender (OR = 0.77, 95 % CI = (0.63, 0.94), *p =* 0.01), and a grade 3 or 4 elevation in lipid measurements (OR = 0.68, 95 % CI = (0.54, 0.86), *p <* 0.01) were associated with a decreased risk of gap between lipid measures, and a grade 3 or 4 elevation in ALT/AST measurement was associated with a decreased risk of a gap between ALT/AST measurements (OR = 0.64, 95 % CI = (0.44,0.93), *p =* 0.02). Results were similar for gaps in excess of 18 months; with the exception that Black race and Grade 3 or 4 levels were no longer significantly associated with gaps in lipid measurements (data not shown).

**Table 4:** Multivariable Generalized Estimating Equation (GEE) models of a gap of 12 months between laboratory measurements

	Liver (AST/ALT)			Renal (Creatinine)			Metabolic (Lipids)		
	(*N =* 3343)			(*N =* 2955)			(*N =* 2838)		
	Odds ratio	95 % CI	*p-*value	Odds ratio	95 % CI	*p-*value	Odds ratio	95 % CI	*p-*value
Province									
British Columbia	Ref			Ref			Ref		
Ontario	1.03	(0.82,1.30)	0.79	0.95	(0.71,1.28)	0.74	2.78	(2.16,3.58)	<0.0001
Quebec	0.99	(0.74,1.32)	0.94	1.18	(0.86,1.61)	0.31	2.56	(1.96,3.33)	<0.0001
Age at cART initiation (per 10 years)	0.77	(0.70,0.84)	<0.0001	0.73	(0.66,0.81)	<0.0001	0.87	(0.80,0.96)	<0.01
Male	0.92	(0.74,1.15)	0.46	0.97	(0.74,1.28)	0.84	0.77	(0.63,0.94)	0.01
Race									
Caucasian	Ref			Ref			Ref		
Black	1.43	(1.05,1.96)	0.02	1.09	(0.69,1.73)	0.71	1.78	(1.29,2.45)	<0.001
Aboriginal	1.09	(0.73,1.64)	0.66	0.80	(0.48,1.33)	0.38	1.22	(0.75,1.96)	0.42
Other	0.52	(0.35,0.75)	<0.001	0.55	(0.35,0.85)	<0.01	0.92	(0.65,1.30)	0.62
Unknown	1.18	(0.92,1.52)	0.19	0.90	(0.69,1.18)	0.44	1.10	(0.90,1.35)	0.36
HCV and IDU status									
HCV- non-IDU	Ref			Ref			Ref		
HCV+ non-IDU	0.98	(0.67,1.43)	0.92	0.83	(0.52,1.33)	0.44	0.72	(0.50,1.03)	0.07
HCV- IDU	1.02	(0.65,1.60)	0.92	1.04	(0.59,1.82)	0.89	0.86	(0.51,1.45)	0.58
HCV+ IDU	2.21	(1.73,2.82)	<0.0001	1.88	(1.40,2.53)	<0.0001	1.70	(1.30,2.23)	<0.001
Unknown	1.15	(0.88,1.49)	0.30	1.47	(1.12,1.93)	0.01	0.55	(0.44,0.68)	<0.0001
Grade 3 or 4 elevation at previous visit	0.64	(0.44,0.93)	0.02	1.74	(0.51,5.96)	0.38	0.68	(0.54,0.86)	<0.01
VL < 50 copies/mL at gap start	0.67	(0.59,0.77)	<0.0001	0.63	(0.52,0.75)	<0.0001	0.82	(0.72,0.93)	<0.01
CD4 count at gap start									
<200 cells/mm^3^	Ref			Ref			Ref		
200-350 cells/mm^3^	0.92	(0.77,1.10)	0.35	1.07	(0.85,1.35)	0.55	1.02	(0.87,1.20)	0.80
350-500 cells/mm^3^	0.88	(0.72,1.07)	0.18	1.08	(0.83,1.39)	0.58	1.11	(0.93,1.33)	0.26
>500 cells/mm^3^	1.03	(0.84,1.26)	0.76	1.26	(0.97,1.63)	0.09	1.12	(0.93,1.35)	0.23
cART initiation year									
2000	Ref			Ref			Ref		
2001-2005	0.56	(0.45,0.70)	<0.0001	0.58	(0.44,0.77)	0.0001	0.83	(0.64,1.08)	0.16
>2005	0.37	(0.29,0.48)	<0.0001	0.39	(0.28,0.54)	<0.0001	0.72	(0.55,0.94)	0.02

*IDU* injection drug use as risk factor for HIV acquisition, *HCV*+ positive for Hepatitis C, *VL* viral load

## Discussion

In this cohort of people infected with HIV from across Canada who had initiated cART since 2000, rates of laboratory test measurement differed by age, race and HIV risk factor despite access to publicly funded health care even after adjusting for rate of CD4 measurement as a surrogate of engagement in care. Persons with HIV-hepatitis C co-infection and with a history of IDU had significantly lower rates of measurement of these tests and were more likely to have a 12 month gap between measurements than participants without history of IDU whether or not they were HIV mono- or HIV-hepatitis C co-infected. However, hepatitis C co-infected participants without a history of IDU were not monitored less frequently than HIV mono-infected individuals, in concordance with clinical guidelines [5, 9].

Our results are similar to those of a multi-site study from the United States of HIV-positive individuals initiating cART, where older age, lower CD4 count, an AIDS diagnosis, later year of cART initiation, boosted PI-based cART regimens and Abacavir use were associated with shorter times to both the first laboratory test and repeated laboratory tests [11]. As in our study, liver enzyme and renal function measurements were more frequent than lipid measurements [11]. However, the study by Yanik *et al* observed higher rates of liver enzyme and renal function measurement than our study (with annual rates of 5.14 within the first 6 months and 3.39 between 6 and 36 months for liver enzymes, and 5.00 and 3.36, respectively, for renal function), but with lower rates of lipid testing [11]. The differences in rates of measurement may be due to study design. Yanik *et al* [11] included participants with at least one laboratory measurement and censored patients at the time of treatment switch or discontinuation and occurrence of abnormal laboratory result, resulting in a median duration of follow-up of 11 months. In our analyses, we counted only one measurement per month to avoid inflated rates due to repeat testing during hospitalization. As in Yanik’s study, we noted slightly higher rates of measurement in the first year after initiation of cART.

In our previous work examining factors associated with rates of viral load (VL) measurement among CANOC participants, geographic region, HIV risk factor, age, year of cART initiation, type of cART regimen, being in the first year of cART, AIDS defining illness and whether or not the previous VL was below the limit of detection were associated with lower rates of VL measurement and gaps in VL measurement of more than 9 months [12]. We have also reported findings from a study of HIV-positive individuals in Ontario, Canada, wherein younger individuals, injection drug users and residents of Toronto had lower rates of VL measurement [13].

Our observations within the current study suggest that challenges in HIV care engagement among people with a history of IDU may considerably limit the ability to follow clinical guidelines for laboratory testing in this population. Previously we have shown that participants with a history of IDU in CANOC were more likely to be suboptimally engaged in HIV care [14], consistent with our present finding of lower rates of laboratory monitoring in this subpopulation. A review paper by Wood *et al* [15] found barriers to care for IDUs included psychiatric illness, financial constraints inhibiting travel to and from clinic, physician perceptions and inexperience with patients with substance use issues, incarceration, and homelessness. Wood *et al* also found that hepatitis C co-infection was associated with less treatment access for IDU, consistent with our findings that hepatitis C co-infected IDU had the lowest rates of laboratory marker measurement and were significantly more likely to have clinically important gaps in measurement than both HIV mono-infected and hepatitis C co-infected non-IDU, and even HIV mono-infected IDU.

The EACS and BC CfE guidelines specifically suggest increased frequency of monitoring for liver and renal function abnormalities for those co-infected with hepatitis C [5, 9]. As such, the disparity in the frequency of clinical monitoring of laboratory markers between hepatitis C co-infected participants with a history of IDU and those without a history of IDU is concerning. Although hepatitis C acquisition has occurred among people who do not inject drugs [16, 17] and such individuals were not at increased risk of gaps in measurement, the majority of HIV-hepatitis C co-infected individuals have IDU as a risk factor. These individuals have elevated risk of liver and kidney disease, yet are monitored less frequently for the development of such comorbid conditions.

When interpreting studies of liver or renal toxicity or the development of comorbid conditions, differential monitoring needs to be considered, as abnormalities are likely to be detected sooner in individuals monitored more frequently [18]. If monitoring patterns are informative, estimates of the association of covariates on rates of adverse events may be biased [18]. Where appropriate, analyses which adjust for differential rates of measurement should be employed; these include marginal structural models for dynamic observation plans [19], interval censoring methods [18], and discrete time survival methods [20]. Explicit discussion of bias introduced from differential monitoring should be discussed if technical limitations preclude the use of these methods.

Strengths of this analysis include the size and diversity of the CANOC cohort, which captures approximately half of the HIV-positive individuals who have initiated cART since 2000 in Canada [10]. The publicly funded health care setting allowed us to assess factors associated with rates of measurement of laboratory markers in the absence of financial barriers directly related to the laboratory test and incurred by the patient. Nevertheless, there are potential limitations that merit consideration which relate to potential information bias. We did not have access to data on risk factors for comorbid conditions such as family history of illness, smoking and body mass index, all of which would influence clinical decision-making regarding the frequency of laboratory monitoring. Further, as some CANOC sites specialize in HIV care, our rates of laboratory monitoring may be underestimated because some patients may be monitored by their primary care physicians. Analyses of data from both HIV care sites and primary care sites would allow a more complete picture of monitoring of comorbidities. Nevertheless, we do not believe that such absence of data would affect our conclusions as Aboriginal peoples and IDU also face barriers to accessing primary care [21–23].

## Conclusions

Despite a publicly funded health care setting, there were disparities in the frequency of liver, renal, and metabolic function laboratory monitoring among subpopulations of HIV-positive individuals. Liver enzymes were more commonly and routinely measured than renal function and lipid tests, and frequency of monitoring differed by age, race, HIV risk factor, and history of hepatitis C co-infection. In particular, people with hepatitis C co-infection and a history of IDU had the lowest rates of laboratory marker measurement despite being at higher risk for cirrhosis and end-stage kidney disease. Further research should evaluate the clinical impact of delayed detection of laboratory abnormalities on the development of comorbid conditions.

### Meetings and conference presentations

The data summarized in this paper were presented in part at the 23^rd^ Canadian Conference on HIV/AIDS Research, St. John’s, Canada, 1-4 May 2014 (Abstract P041).

## Abbreviations

AIDS: Acquired immune deficiency syndrome
ALT: Alanine transaminase
AST: Aspartate transaminase
BC: British Columbia
BC CfE: British Columbia Centre for Excellence in HIV/AIDS
BHIVA: British HIV Association
CANOC: Canadian Observational Cohort
cART: Combination antiretroviral therapy
CI: Confidence interval
DHHS: Department of Health and Human Services
EACS: European AIDS Clinical Society
GEE: Generalized estimation equation
HDL: High-density lipoprotein
HIV: Human immunodeficiency virus
IDU: Injection drug use
IQR: Interquartile range
LDL: Low-density lipoprotein
MSM: Men having sex with men
OR: Odds ratio
RR: Rate ratio
ULN: Upper limit of normal
